# A Rare Cause of Left Ventricular Mass: Cardiac Hemangioma

**DOI:** 10.4274/balkanmedj.2017.1334

**Published:** 2018-07-24

**Authors:** Cihan Altın, Hakan Güllü, Ziya Gökalp Bilgel, Mustafa Yılmaz, Tuba Canpolat, Öner Gülcan

**Affiliations:** 1 Department of Cardiology, Başkent University of School of Medicine, İzmir, Turkey; 2 Department of Cardiology, Başkent University of School of Medicine, Adana, Turkey; 3 Department of Pathology, Başkent University of School of Medicine, Adana, Turkey; 4 Department of Cardiovascular Surgery, Başkent University of School of Medicine, Adana, Turkey

Cardiac hemangiomas are uncommon tumors of the heart and have been primarily documented as isolated case reports in the literature (1,2,3,4,5). Herein, we present a case of a 56-year-old female having an incidentally identified left ventricular mass. She had no history of cardiac or systemic disease and no clinical symptoms other than mild palpitation. Cardiac imaging tools revealed a hyperechoic, rounded, mobile mass with clear borders, originating from the anteroseptal wall of the left ventricle and protruding into the left ventricular cavity (Figure 1a-c). Preoperative coronary angiography demonstrated that the tumor was fed by septal coronary arteries and the contrast medium was pooling within the tumor area creating the “*tumor blush*” appearance (Figure 1d). The patient was successfully operated, and the pedunculated mass measuring 2.3×1.5×1.0 cm was completely excised (Figure 1e). The pathological diagnosis was reported to be hemangioma (Figure 2). She had an uneventful recovery without any complication and was discharged on the postoperative 5^th^ day. Informed consent was obtained from the patient to publish this case report. 

As echocardiography has become an easily accessible tool, the incidence rates of cardiac masses have dramatically increased. According to surgical and autopsy reports, the incidence of primary cardiac tumors has been reported to be 0.001%–0.5%, and approximately 75% of them are benign (1,2). Cardiac hemangioma, a vascular tumor of the heart, accounts for 5% of these benign cardiac tumors (2,3,4). In the majority of cases, they do not cause any symptom and are diagnosed incidentally, whereas some tumors can be symptomatic. Symptoms can arise as a consequence of the following tumor evolution: compression, infiltration, rupture, embolization, and growth. Life-threatening complications, including outflow tract obstruction, coronary insufficiency, heart failure, pericardial tamponade, thromboembolia, dysrhythmia, and even sudden cardiac death, can be noticed (1,2,3,4,5). Clinical presentation depends on the location, size, and expansion of the tumor. Since the natural course of these tumors is quite variable and unpredictable, surgical resection is the treatment of choice (1,2). Patients with a resectable cardiac hemangioma have good surgical outcome and prognosis. Although recurrence has been reported in limited cases (4), postoperative long-term outcome is favorable. In our case, there was no evidence of recurrence at the 10^th^-month of follow-up.

In conclusion, among primary cardiac tumors, hemangiomas are relatively rare. Nevertheless, clinicians should consider cardiac hemangioma in the differential diagnosis of cardiac masses, especially in patients with a typical angiographic “*tumor blush*” appearance, which demonstrates the vascular nature of the tumor.

## Figures and Tables

**Figure 1:**
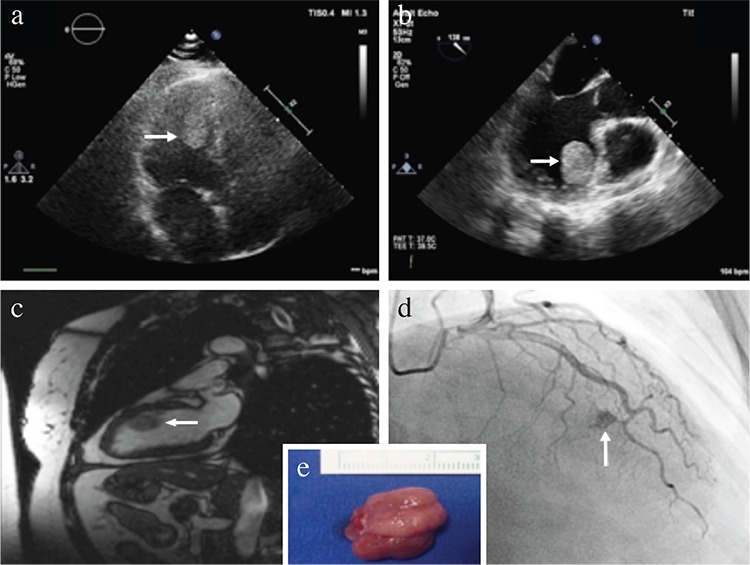
Preoperative transthoracic echocardiography; apical four-chamber view showing a hyperechoic, round, mobile mass (arrow) (a). Transesophageal echocardiography revealed a homogenous, round, pedunculated mass (arrow) originating in the anteroseptal wall of the left ventricle (b). A round, hyperintense mass (arrow) originating in the anteroseptal wall of the left ventricle is seen on the two-chamber white blood cardiac magnetic resonance imaging (c). Preoperative coronary angiography demonstrated the characteristic finding of cardiac hemangioma “tumor blush” (arrow), which highlights the vascular nature of the tumor (d). Image of the excised cardiac mass measuring 2.3×1.5×1.0 cm (e).

**Figure 2:**
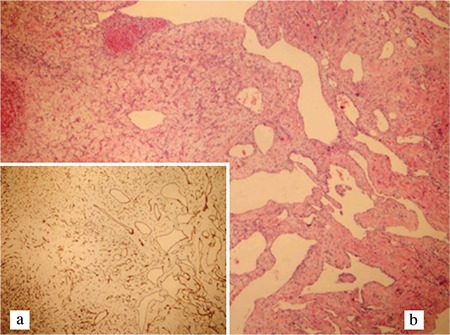
Diffuse staining of the vascular endothelial cells with CD34 antibody at ×100 magnification (a), Variations in size and dilated vessels in an edematous stroma; H&E staining, ×100 magnification (b).
